# Perceived burden and family functioning among informal caregivers of individuals living with schizophrenia in Tanzania: a cross-sectional study

**DOI:** 10.1186/s12888-021-03560-0

**Published:** 2022-01-04

**Authors:** Rosarito Clari, Jennifer Headley, Joseph Egger, Praxeda Swai, Paul Lawala, Anna Minja, Sylvia Kaaya, Joy Noel Baumgartner

**Affiliations:** 1 Duke Global Health Institute, Duke University, Durham, NC USA; 2 School of Medicine, Muhimbili University of Health and Allied Sciences, Dar es Salaam, Tanzania; 3 Mirembe National Mental Health Hospital, Dodoma, Tanzania; 4 School of Social Work, University of North Carolina at Chapel Hill, Chapel Hill, NC USA

**Keywords:** Caregiver burden, Caregiving, Family functioning, Schizophrenia, Psychotic disorders

## Abstract

**Background:**

Globally, families play a critical role in providing care and support for persons living with schizophrenia. It is important to identify potentially modifiable factors that influence perceived caregiver burden in order to properly address the needs of caregivers. This is especially relevant in low-resource settings where psychiatric services are scarce and interventions could be most effective if targeted to both the individual living with schizophrenia and their caregiver. This study examines correlates of perceived burden among informal caregivers of individuals with schizophrenia in Tanzania, in particular, the association between burden and caregiver-reported family functioning.

**Methods:**

This study used baseline data from an individually randomized controlled trial with 65 pairs of individuals with schizophrenia and their informal caregivers in Dar es Salaam and Mbeya, Tanzania. Caregiver burden was measured using the Burden Assessment Scale. Univariable and multivariable regression analyses were performed to determine the relationship between caregiver burden and family functioning and to explore other correlates of burden.

**Results:**

Sixty-three percent of caregivers reported experiencing high burden as a result of caring for a relative with schizophrenia. Multivariable regression analyses revealed that poor family functioning and the caregiver being employed were associated with high caregiver burden, while higher levels of hopefulness in the caregiver was associated with low caregiver burden.

**Conclusion:**

Caregivers who were employed, reported poor family functioning, and/or had low levels of hopefulness were more likely to perceive high caregiver burden. Future interventions aiming to reduce caregiver burden may benefit from improving family functioning and nurturing hope among caregivers of individuals living with schizophrenia. Policies and programs should be cognizant of the needs of caregivers that work in addition to providing care for a relative with schizophrenia in order to better support them.

**Supplementary Information:**

The online version contains supplementary material available at 10.1186/s12888-021-03560-0.

## Introduction

In low- and middle-income countries, family involvement in psychiatric care plays a crucial role in the recovery of individuals living with schizophrenia due to a global shift away from institutionalized psychiatric care and limited availability of community-based psychiatric services and mental health resources [1, 2]. There is extensive evidence from high-income countries indicating that informal caregiving for an individual with schizophrenia is associated with poor mental, physical, social, and financial outcomes for the caregiver [3–7]. More recently, studies conducted in sub-Saharan Africa have shown comparably high levels of caregiver burden, impacting similar domains. For example, a study in Nigeria found that caregivers of individuals with schizophrenia are at risk for mental disorders themselves [8]. Moreover, a systematic review of studies done in multiple African countries, including Ghana, Ethiopia, and South Africa, reported moderate to severe caregiver burden characterized by financial constraint, productivity loss, and lost employment [9]. In addition to severe psychological and financial burden, qualitative interviews conducted with caregivers of individuals with schizophrenia in Zimbabwe revealed physical and social burden associated with caregiving [10].

Caregiver burden refers to the strain endured by a person who cares for a chronically ill individual [11]. Although there has been much debate in the literature regarding the dimensions and attributions of the construct, a distinction has been made between objective and subjective burden [11]. Objective burden includes concrete costs to the caregiver, such as time and finances devoted to care. Subjective burden, in contrast, refers to the extent to which the caregiver feels the burden of care [12]. It is important to draw attention to the negative connotation of the concept of burden, which obscures any positive experiences related to care. In fact, there is evidence suggesting that caregivers become more sensitive to persons with disabilities, gain insight into their life priorities, and experience a deeper sense of inner strength as a result of caring for an individual with schizophrenia [13]. We should therefore clarify that the present study is concerned with the negative effects of caregiving.

There are certain socio-demographic and illness-related factors that can render caregivers of individuals with schizophrenia more vulnerable to experiencing burden. Likewise, there are factors that can be protective against the consequences of the illness’s impact on the caregiver. Studies on factors associated with burden among families or caregivers of a person living with schizophrenia in Africa are few and sparse. The literature suggests that caregivers who are female, older, and with lower education levels are more likely to experience burden, and that illness-related risk factors (i.e., greater illness severity, longer duration of illness) can exacerbate the experience of burden [8, 14–17]. When it comes to potential protective factors, higher levels of income and social support have been linked to the caregiver’s well-being [15, 18].

An important goal in the field of global mental health is to develop evidence-based interventions that target not only the individual living with schizophrenia but the family as a unit [2]. Given the significant involvement of family members in psychiatric care, it is imperative to address the needs of caregivers in conjunction with those of the individuals living with schizophrenia. In order to develop such family-focused interventions, however, we need to gain a deeper knowledge of the factors that are associated with perceived caregiver burden.

In Tanzania, to the best of our knowledge, there has been no research on this particular area. Considerable work is needed to elucidate specific factors that influence the extent of caregiver burden to recommend culturally appropriate areas for supportive interventions. Studies conducted in urban Tanzania found that poor family and social support are significant determinants for relapse in schizophrenia [19, 20]. A better understanding of these factors can have important implications for the caregiver’s well-being as well as for the recovery and overall quality of life of the individual living with schizophrenia.

Among the factors that could impact perceived burden of caring for someone living with schizophrenia is family functioning [4, 21]. Family functioning refers to the ways in which relationships operate within the family and is believed to be central to the welfare of all family members; dysfunctional family processes can lead to psychological problems and conversely, positive and supportive family processes can facilitate therapeutic change [22]. Nevertheless, family functioning has often been overlooked in the literature, particularly in low-resource settings. The few studies that evaluated family functioning in relation to caregiver burden have been conducted in high-income countries [4, 21]. In China, Yu et al. (2017) demonstrated that higher family functioning, among other factors, was an important correlate of decreased family burden. In a study conducted in Spain, Ribé et al. (2018) showed that caregivers of individuals with schizophrenia with low levels of caregiver burden and high levels of family functioning tended to report better quality of life than their counterparts. The present study aimed to address this gap in the literature by examining caregiver burden and family functioning in a sub-Saharan African country.

The purpose of this study was to examine the levels of perceived burden among caregivers of individuals with schizophrenia and its relationship to family functioning in Dar es Salaam and Mbeya regions in Tanzania. In addition, we aimed to identify socio-demographic factors of both individuals living with schizophrenia and caregivers as well as illness-related factors that are associated with perceived caregiver burden among the caregiver study population. This research study represents an important first step towards understanding perceived caregiver burden and tailoring interventions to improve outcomes for individuals with schizophrenia and their relatives.

## Methods

### Study overview

This study uses pre-intervention baseline data from a pilot individually randomized group treatment (IRGT) trial (ClinicalTrials.gov # NCT04013932) to assess the feasibility and acceptability of a culturally tailored Family Psychoeducation intervention for individuals with psychotic disorders and their relatives in Tanzania.

### Study setting

The clinical trial was carried out through a partnership between Duke University, Columbia University, and Muhimbili University of Health and Allied Sciences (MUHAS) in two sites located in the regions of Dar es Salaam and Mbeya, Tanzania. The first study site was Muhimbili National Hospital (MNH), the national referral hospital in Dar es Salaam, which serves local area residents as well as referrals from across the country. The Department of Psychiatry at MNH provides inpatient and outpatient care and its staff includes psychiatrists, psychiatric nurses, social workers, occupational therapists, and psychologists. Although general mental health education classes are offered at MNH for outpatients and their families, outpatient psychiatric services largely focus on medication management.

The second study site was Mbeya Zonal Referral Hospital (MZRH), which is located in Mbeya city. It is the only referral facility in the southern part of the country with a total population of approximately 2 million. The Psychiatry and Mental Health Unit at MZRH is comprised of one psychiatrist, general practitioners, psychiatric nurses, and social workers. Similar to MNH, MZRH may offer ad hoc mental health education and family counseling sessions but does not offer any structured and evidence-based routine psychosocial services for adults with psychotic disorders.

### Participants

A total of 66 pairs of individuals with schizophrenia and their matched caregivers were consented and enrolled in the study. Inclusion criteria for individuals with schizophrenia included attending outpatient psychiatric services at MNH or MZRH, having an ICD-10 (International Classification of Disease) diagnosis of a non-organic psychotic disorder, ages 18–50 years at the time of informed consent, and hospitalization or non-hospitalized relapse within the past year. For matched caregivers/relatives, the inclusion criteria included ages 18 years or older at the time of the informed consent and the individual with schizophrenia agreeing to be partnered with this person for the duration of the clinical trial.

### Procedures

Data collection for the present study took place at both study sites in September and October of 2019. Individuals with schizophrenia who attended outpatient services at MNH or MZRH and expressed an interest in the study were screened for eligibility. Every outpatient then identified a caregiver, usually a relative, who could participate in the study. If both were eligible, the pair provided informed consent after being given thorough information about the study. All participants with psychotic disorders had to be stable at the time of the informed consent process. The study psychiatrists were responsible for determining whether the participant was stable and had the competence and capacity to consent to research participation. All participants were paid 7500 Tsh (~US $3.50) for travel and subsistence costs related to their study attendance. Study visits were carried out at office facilities within MNH or MZRH. All assessments were done in Kiswahili, the official language in Tanzania. Data were collected electronically on encrypted tablets through the online REDCap server.

### Ethical approval

All study procedures were approved by the ethical review boards at Duke University Medical Center (Protocol No. Pro00094163), Muhimbili University of Health and Allied Sciences (Ref No. DA.282/298/0 I.C), Mbeya Zonal Referral Hospital (Ref No. SZEC-2./39/R.E IV 11–13), and the Tanzanian National Institute for Medical Research (Ref No. NIMRJHQ/R.8a/Vol. IX/3156).

### Measures

Data for the present study utilized measures from both informal caregivers and individuals living with schizophrenia. All scales used in the study underwent the World Health Organization’s four-step process for translation and cultural validation, namely, forward-translation, back-translation, pre-testing, and finalization with expert consensus [23].

#### Measures for informal caregivers

**Fig. 1:**
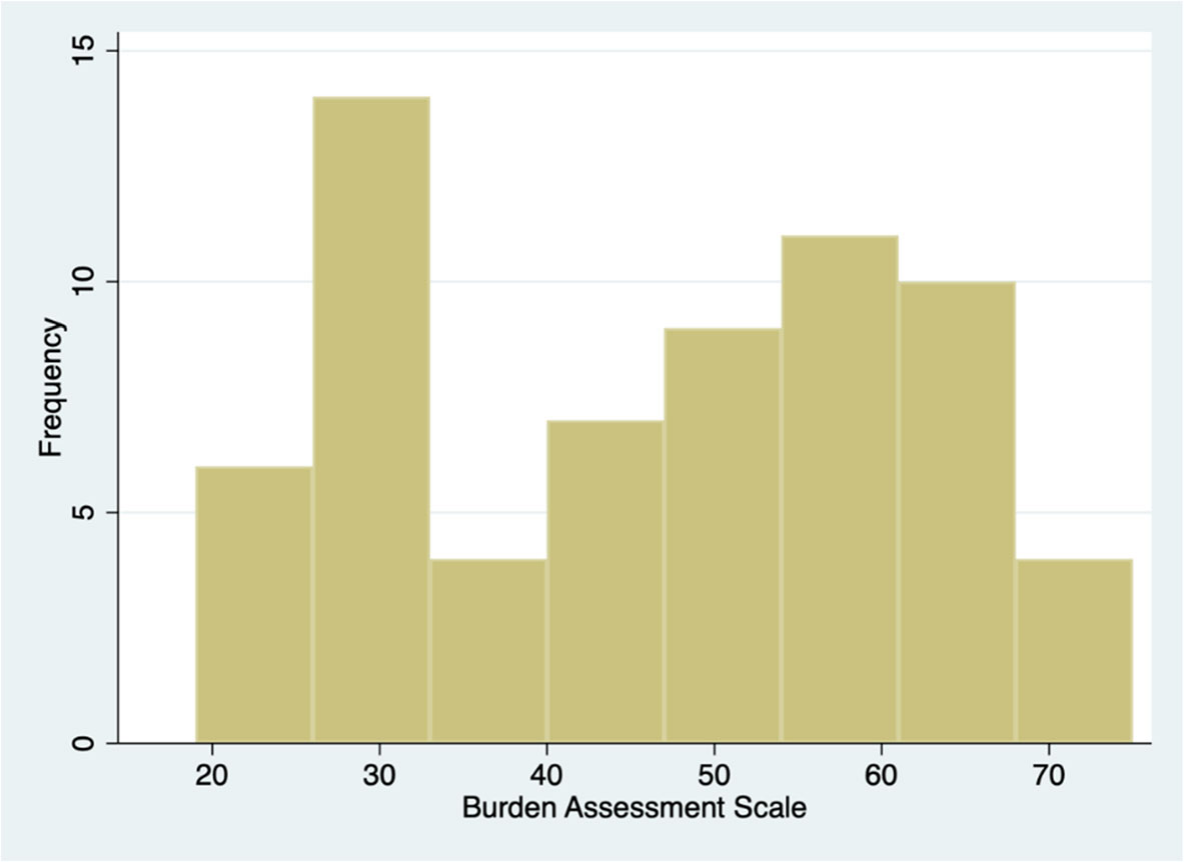
Caregiver Burden Score Distribution

The main outcome variable, the Burden Assessment Scale (BAS), was completed by the matched caregiver as a measure of perceived burden [24]. The scale consists of 19 items assessing perceptions of burden associated with providing support to a relative with mental illness. Items 1–10 measure objective consequences, such as financial difficulties and limitations on personal activity. Items 11–19 assess subjective consequences, including shame, stigma, and resentment. Each item is rated on a 4-point Likert scale (1 = not at all; 4 = a lot). A sum score was computed ranging from 19 to 76, where higher scores indicated greater levels of caregiver burden. The frequency distribution of the variable was inspected visually and showed a natural break in the data at 40, which was used to dichotomize the variable (Fig. 1). The cut-off point for the BAS for the main analysis was 19–39 for low burden and 40–76 for high burden. As a sensitivity analysis, we also performed analyses of association using another cut-off point, also at a natural break in the data, specifically 19–32 for low burden and 33–76 for high burden. In this study, the Cronbach’s alpha for the BAS was 0.95, indicating high internal consistency or reliability.

The main exposure variable was family functioning as reported by the matched caregiver via the 15-item version of the Systemic Clinical Outcome and Routine Evaluation (SCORE-15) [25]. The SCORE-15 is a questionnaire for completion by family members of individuals engaged in systemic therapy to evaluate family functioning. It has a three-factor structure, which assesses family strengths, difficulties, and communication. Statements about family life are rated on a 5-point Likert scale from 1 (describes us: very well) to 5 (describes us: not at all). The total score ranges from 15 to 75, with a lower score indicating better family functioning. A total average score is used for analysis purposes and ranges from 1 to 5, with the same directionality as the total score. The Cronbach’s alpha of the SCORE-15 was 0.80 in this study, indicating good reliability.

The Hearth Hope Index (HHI) was used to assess hope in caregivers. The HHI contains 12 items and evaluates three factors of hope: temporality and future, positive readiness and expectancy, and interconnectedness [26]. The HHI scores range from 12 to 48, the higher the score the higher the level of hope. The internal consistency of the HHI was very good in this study (Cronbach’s alpha = 0.92).

The Duke University Religion Index (DUREL) is a 5-item scale that was used to measure religiosity in caregivers [27]. The DUREL scale assesses 3 major dimensions of religiosity: organizational religious activity, nonorganizational religious activity, and intrinsic religiosity. According to the developers of the scale, each dimension is meant to be analyzed separately. In this study, we were particularly interested in Intrinsic Religiosity (IR) and, therefore, only focused on that subscale for analyses. The IR dimension contains 3 items that evaluate the degree of personal religious commitment or motivation on a 5-point scale, from 1 (definitely not true) to 5 (definitely true of me). The score range of the IR subscale is 3 to 15, with higher scores indicating higher religious involvement. The Cronbach’s alpha of the DUREL’s IR subscale was 0.61, indicating acceptable reliability.

#### Measures for individuals with schizophrenia

The severity of symptoms was measured using the Positive and Negative Syndrome Scale (PANSS) [28]. The PANSS consists of a structured interview to assess individuals with schizophrenia on 30 items covering positive and negative symptoms as well as general psychopathology. Of the thirty items included in the PANSS, seven constitute a positive scale, seven a negative scale, and the remaining sixteen a general psychopathology scale. Items are rated on a 1 (absent) to 7 (extreme) scale of increasing levels of psychopathology. The Cronbach’s alpha of the PANSS was 0.91 in this study, indicating high internal consistency.

Functioning of the individual with schizophrenia was measured using the World Health Organization Disability Assessment Schedule-Second Version (WHODAS 2.0) [29]. This self-report assessment measures the difficulty the individual has had with performing daily activities over the past 30 days. It consists of 36 Likert-formatted questions, divided into six domains: understanding and communicating, getting around, self-care, getting along with others, life activities, and participation in society. The complex scoring method was used to determine a total score, ranging from 0 (no disability) to 100 (full disability). The Cronbach’s alpha of the WHODAS 2.0 was 0.96 in this study, indicating high reliability.

The General Self-Efficacy Scale (GSE) was used to assess the perceived ability to manage stressful situations of individuals living with schizophrenia [30]. Respondents indicate their level of agreement with 10 items (e.g., “I can solve most problems if I invest the necessary effort”) on a Likert-type scale ranging from 1 (not true at all) to 4 (exactly true). The total score is calculated by summing all items and ranges between 10 and 40, with higher scores indicating more self-efficacy. The Cronbach’s alpha of the GSE was 0.89 in this study.

The Internalized Stigma of Mental Illness (ISMI) scale was utilized to measure subjective experience of stigma in individuals living with schizophrenia [31]. The ISMI contains 29 items with a 4-point Likert scale and evaluates five areas (i.e., subscales) of self-stigma: alienation, stereotype endorsement, perceived discrimination, social withdrawal, and stigma resistance. In this study, the internal consistency of the scale was very good (Cronbach’s alpha = 0.92).

Instrumental support was assessed using a measure from PROMIS v2.0 (Patient-Reported Outcomes Measurement Information System). All PROMIS measures were developed and validated by a National Institutes of Health (NIH) working group [32]. The Instrumental Support scale comprises 11 items asking whether respondents have someone who could assist with various daily tasks. The internal consistency of the scale was very high (Cronbach’s alpha = 0.96).

Self-reported socio-demographic characteristics of informal caregivers and individuals with schizophrenia included age, sex, education, relationship status, employment situation, and financial contribution to the household. Information regarding the relationship of the informal caregiver to the individual with schizophrenia, including whether they were living together, was also collected. Lastly, clinical characteristics were obtained from the outpatients’ medical charts and included the year of illness onset, which was used to calculate the length of illness.

### Data management and analysis

Descriptive statistics are presented for the full sample (66 pairs). However, due to incomplete data for one caregiver, the regression analyses included 65 individuals with schizophrenia and 65 matched caregivers, excluding the pair with missing data. Missing values in the SCORE-15, HHI, and WHODAS were handled using mean imputation—the imputed value being the average of all observed responses within the same domain for that participant—as per the recommendation of the developers of the scales [26, 29, 33]. Univariable and multivariable logistic regression models were fitted to data to explore potential correlates of caregiver burden, including family functioning.

The method used to select variables for the multivariable regression was data-driven but also consistent with a priori theory. Given the small sample size and a lack of a strong understanding of the relationship between some of the variables, the final model included variables that were found to be associated with caregiver burden, as well as family functioning, in univariable regression analyses, using a type I error rate of 5%. Previous research indicates that there is no relationship between family functioning and sex of the individual with schizophrenia [34, 35]; as a result, sex of the individual with schizophrenia was not included in the final model. STATA 16.0 statistical software was used to perform all analyses [36].

## Results

### Participant characteristics

The socio-demographic and clinical characteristics of individuals living with schizophrenia stratified by sex are presented in Table 1. The average age of individuals living with schizophrenia was 33 years old [Standard Deviation (SD) = 8.2]. A large majority reported being single (66.7%) and completing secondary education or obtaining a higher degree (62.1%). Whereas 57.6% of individuals with schizophrenia indicated having worked in the past 3 months, only 34.9% reported having contributed financially to their household. The average length of illness was 9 years, with a minimum of less than a year and a maximum of 29 years. The average disability score in the WHODAS was 37.5 (SD = 20.6). The mean score of the severity of symptoms was 11.0 (SD = 4.3) for positive PANSS scale score, 11.1 (SD = 4.7) for negative PANSS scale score, 23.8 (SD = 7.7) for general PANSS scale score, and 45.9 (SD = 14.5) for total PANSS score.

**Table 1:** Characteristics of Individuals Living with Schizophrenia, Stratified by Sex

	Total	Men	Women
	(*N* = 66)	(*N* = 44)	(*N* = 22)
**Age Categorized**			
< = 24	10 (15.2%)	7 (15.9%)	3 (13.6%)
25–34	27 (40.9%)	20 (45.5%)	7 (31.8%)
35–50	29 (43.9%)	17 (38.6%)	12 (54.6%)
**Age, in years**			
Mean (SD)	33.0 (8.2)	32.6 (8.2)	33.9 (8.4)
Min, Max	18, 50	18, 49	21, 50
**Relationship Status**			
Partnered, living together	10 (15.2%)	7 (15.9%)	3 (13.6%)
Partnered, not living together	12 (18.2%)	4 (9.1%)	8 (36.4%)
Single, not partnered	44 (66.7%)	33 (75.0%)	11 (50.0%)
**Educational Level**			
Primary or less	25 (37.9%)	15 (34.1%)	10 (45.5%)
Secondary or higher	41 (62.1%)	29 (65.9%)	12 (54.6%)
**Worked in the Past 3 Months**			
No	28 (42.4%)	16 (36.4%)	12 (54.6%)
Yes	38 (57.6%)	28 (63.6%)	10 (45.5%)
**Financial Contribution to Household**			
No	43 (65.2%)	26 (59.1%)	17 (77.3%)
Yes	23 (34.9%)	18 (40.9%)	5 (22.7%)
**Length of Illness, in years**			
Mean (SD)	9.1 (8.1)	9 (7.5)	9.4 (9.3)
Min, Max	0, 29	0, 29	0, 26
**Disability (WHODAS)**			
Mean (SD)	37.5 (20.6)	37.2 (20.5)	38.1 (21.3)
Min, Max	0, 83.7	0, 82.6	4.3, 83.7
**Positive PANSS Scale Score**			
Mean (SD)	11.0 (4.3)	11.9 (4.8)	9.2 (2.2)
Min, Max	7, 26	7, 26	7, 15
**Negative PANSS Scale Score**			
Mean (SD)	11.1 (4.7)	11.6 (5.2)	9.9 (3.3)
Min, Max	7, 30	7, 30	7, 20
**General PANSS Scale Score**			
Mean (SD)	23.8 (7.7)	24.5 (8.7)	22.6 (5.3)
Min, Max	16, 60	16, 60	16, 35
**Total PANSS Score**			
Mean (SD)	45.9 (14.5)	48.0 (16.4)	41.7 (8.9)
Min, Max	30, 103	30, 103	30, 67

*WHODAS* World Health Organization Disability Assessment Schedule, *PANSS* Positive and Negative Syndrome Scale

Table 2 shows socio-demographic and other characteristics of informal caregivers stratified by sex. The average age of caregivers was 48.8 years (SD = 13.1). More than half of the caregivers (51.5%) reported currently being in a relationship and living with their partner. Most caregivers (63.6%) indicated that the highest level of education attained was primary school. Fifty-three percent of caregivers reported having worked in the past 3 months and a large majority (74.2%) had financially contributed to their household. Informal caregivers were mostly parents of the individual living with schizophrenia (48.5%) and lived in the same household (84.9%). The average religiosity score from DUREL’s IR subscale was 14.2 (SD = 1.1) and the average hope score in the HHI was 38.7 (SD = 6.7) for caregivers. The main independent variable, family functioning, had a mean of 2.4 (SD = 0.6) in the SCORE-15. Lastly, the average caregiver burden reported in the BAS was 45.8 (SD = 15.7), with most caregivers (63.1%) falling into the high burden category.

**Table 2:** Characteristics of Informal Caregivers, Stratified by Sex

	Total	Men	Women
	(*N* = 66)	(*N* = 23)	(*N* = 43)
**Age Categorized**			
< = 24	1 (1.5%)	1 (4.4%)	0 (0.0%)
25–49	32 (48.5%)	13 (56.5%)	19 (44.2%)
50+	33 (50.0%)	9 (39.1%)	24 (55.8%)
**Age, in years**			
Mean (SD)	48.8 (13.1)	47.0 (15.3)	49.7 (11.9)
Min, Max	21, 72	21, 72	25, 70
**Relationship Status**			
Partnered, living together	34 (51.5%)	14 (60.9%)	20 (46.5%)
Partnered, not living together	9 (13.6%)	4 (17.4%)	5 (11.6%)
Single, not partnered	23 (34.9%)	5 (21.7%)	18 (41.9%)
**Educational Level**			
Primary or less	42 (63.6%)	14 (60.9%)	28 (65.1%)
Secondary or higher	24 (36.4%)	9 (39.1%)	15 (34.9%)
**Worked in the Past 3 Months**			
No	31 (47.0%)	11 (47.8%)	20 (46.5%)
Yes	35 (53.0%)	12 (52.2%)	23 (53.5%)
**Financial Contribution to Household**			
No	17 (25.8%)	3 (13.0%)	14 (32.6%)
Yes	49 (74.2%)	20 (87.0%)	29 (67.4%)
**Relationship to Individual with Schizophrenia**			
Spouse/Partner	7 (10.6%)	2 (8.7%)	5 (11.6%)
Child	2 (3.0%)	1 (4.4%)	1 (2.3%)
Parent	32 (48.5%)	7 (30.4%)	25 (58.1%)
Sibling	12 (18.2%)	6 (26.1%)	6 (14.0%)
Other relatives	11 (16.7%)	6 (26.1%)	5 (11.6%)
Friend	2 (3.0%)	1 (4.4%)	1 (2.3%)
**Living Together**			
No	10 (15.2%)	4 (17.4%)	6 (14.0%)
Yes	56 (84.9%)	19 (82.6%)	37 (86.1%)
**Religiosity (IR Subscale of DUREL)**			
Mean (SD)	14.2 (1.1)	14.3 (1.1)	14.2 (1.2)
Min, Max	10, 15	11, 15	10, 15
**Hope (HHI) †**			
Mean (SD)	38.7 (6.7)	39.0 (6.6)	38.6 (6.8)
Min, Max	14, 48	25, 48	14, 48
**Family Functioning (SCORE-15) †**			
Mean (SD)	2.4 (0.6)	2.3 (0.6)	2.5 (0.6)
Min, Max	1.1, 3.6	1.3, 3.5	1.1, 3.6
**Caregiver Burden (BAS), Continuous †**			
Mean (SD)	45.8 (15.7)	43.2 (17.4)	47.1 (14.8)
Min, Max	19, 75	19, 75	22, 73
**Caregiver Burden (BAS), Categorical †**			
Low Burden	24 (36.9%)	10 (45.5%)	14 (32.6%)
High Burden	41 (63.1%)	12 (54.6%)	29 (67.4%)

*DUREL* Duke University Religion Index *SCORE-15* Systemic Clinical Outcome and Routine Evaluation, *BAS* Burden Assessment Scale, *HHI* Herth Hope Index

†*N* = 65 (Men = 22, Women = 43)

### Correlates of caregiver burden

The results of univariable logistic regressions of the relationship between caregiver burden and demographic, clinical, and other characteristics, respectively, are presented in Table 3. Regarding the main independent variable, the logistic regression model indicated that lower levels of family functioning, that is higher scores in the SCORE-15, were significantly associated with high caregiver burden using our primary cut-off point of 39/40 [Odds Ratio (OR) = 11.12; 95% Confidence Interval (CI) = 3.22, 38.40]. Other factors that were significantly associated with caregiver burden in univariable analysis were sex of the individual with schizophrenia (OR = 3.91; 95% CI = 1.13, 13.50), caregiver work (OR = 4.68; 95% CI = 1.57, 13.95), and caregiver hope (OR = 0.78; 95% CI = 0.69, 0.89). Individual with schizophrenia being female and caregiver having worked in the past 3 months were associated with high caregiver burden, while higher levels of hope in the caregiver were associated with low caregiver burden.

**Table 3:** Univariable Regression Models for Caregiver Burden (Cut-Off Point of 39/40) on Characteristics of Individuals with Schizophrenia and Informal Caregivers (*N* = 65)

		Univariable Model	
***Characteristics of Individuals with Schizophrenia***	**N**	**Crude OR (95% CI)**	***p*-value**
**Sex**			
Male	43	REF	
Female	22	3.91 (1.13; 13.50)	0.031
**Relationship Status**			
Partnered, living together	10	REF	
Partnered, not living together	11	4.50 (0.63; 32.29)	0.135
Single, not partnered	44	1.59 (0.40; 6.31)	0.511
**Educational Level**			
Primary or less	24	REF	
Secondary or higher	41	0.43 (0.14; 1.29)	0.132
**Worked in the Past 3 Months**			
No	28	REF	
Yes	37	0.39 (0.13; 1.15)	0.087
**Financial Contribution to Household**			
No	43	REF	
Yes	22	0.58 (0.20; 1.66)	0.310
**Age**	65	0.96 (0.90; 1.02)	0.164
**Length of Illness**	65	0.97 (0.91; 1.04)	0.398
**Disability (WHODAS)**	65	1.00 (0.97; 1.02)	0.939
**Self-Efficacy (GSE)**	65	1.03 (0.95; 1.12)	0.528
**Instrumental Support (PROMIS)**	65	1.01 (0.97; 1.06)	0.511
**Internalized Stigma (ISMI)**	65	1.44 (0.51; 4.03)	0.491
**PANSS Positive**	65	0.93 (0.83; 1.05)	0.239
**PANSS Negative**	65	0.92 (0.82; 1.03)	0.139
**PANSS General**	65	0.96 (0.90; 1.03)	0.253
**PANSS Total**	65	0.97 (0.94; 1.01)	0.153
***Characteristics of Informal Caregivers***	**N**	**Crude OR (95% CI)**	***p*-value**
**Sex**			
Male	22	REF	
Female	43	1.73 (0.60; 4.95)	0.310
**Educational Level**			
Primary or less	41	REF	
Secondary or higher	24	0.96 (0.34; 2.73)	0.941
**Worked in the Past 3 Months**			
No	31	REF	
Yes	34	4.68 (1.57; 13.95)	0.006
**Living with individual with schizophrenia**			
No	10	REF	
Yes	55	0.69 (0.16; 2.98)	0.623
**Age**	65	0.98 (0.94; 1.02)	0.377
**Family Functioning (SCORE-15)**	65	11.12 (3.22; 38.40)	< 0.001
**Hope (HHI)**	65	0.78 (0.69; 0.89)	< 0.001
**Religiosity (IR Subscale of DUREL)**	65	0.61 (0.35; 1.08)	0.091

*OR* odds ratio, *CI* confidence interval

*WHODAS* World Health Organization Disability Assessment Schedule, *GSE* General Self-Efficacy scale, *PROMIS* Patient-Reported Outcomes Measurement Information System, *ISMI* Internalized Stigma of Mental Illness scale, *PANSS* Positive and Negative Syndrome Scale, *SCORE-15* Systemic Clinical Outcome and Routine Evaluation, *HHI* Herth Hope Index, *DUREL* Duke University Religion Index

Our sensitivity analysis, performed on all univariable regression analyses using a cut-off point 32/33 for the BAS instead of a cut-off point of 39/40, can be found in Additional file 1. Use of this lower cut-off point re-categorized 4 (6.15%) individuals from low burden to high burden. The association between caregiver burden and family functioning was further from the null (OR = 26.70; 95% CI = 5.52, 129.22), and other relationships were either similar or attenuated, compared to our main analyses.

All variables that were significantly associated with caregiver burden using the primary cut-off point, as well as with family functioning, were included in the multivariable logistic regression. Family functioning, caregiver work, and caregiver hope, respectively, remained significant after controlling for all other factors (Table 4). On adjustment, the effect of family functioning on caregiver burden was significantly attenuated. For every one-point increase on the family functioning measure (i.e., lower family functioning), the odds of experiencing high caregiver burden increases approximately fivefold, holding all other variables constant. The effects of caregiver hope and caregiver work on caregiver burden did not change considerably when adjusting for the other variables. With every one-point decrease in the levels of hope of the caregiver, we observed an 18% increase in the odds of experiencing high caregiver burden, while holding all other variables constant. Caregivers that worked in the past 3 months have almost five times the odds of experiencing high burden than their counterparts.

**Table 4:** Multivariable Regression Model for Caregiver Burden Using Caregiver Characteristics (*N* = 65 pairs)

		Multivariable Model	
***Characteristics of Informal Caregivers***	N	Adjusted OR (95% CI)	***p***-value
**Worked in the Past 3 Months**			
No	31	REF	
Yes	34	4.81 (1.14; 20.26)	0.032
**Family Functioning (SCORE-15)**	65	4.78 (1.19; 19.25)	0.028
**Hope (HHI)**	65	0.82 (0.71; 0.95)	0.008

*OR* odds ratio, *CI* confidence interval

*SCORE-15* Systemic Clinical Outcome and Routine Evaluation, *HHI* Herth Hope Index

## Discussion

The majority of our caregiver sample (63.1%) reported experiencing high burden as a result of caring for a relative living with schizophrenia. This is generally consistent with previous reports of significant burden in informal caregivers in other parts of sub-Saharan Africa, including Ghana and Nigeria [17, 37, 38]. As hypothesized and established in former research, we found that family functioning was an important correlate of caregiver burden [21]. More specifically, lower levels of family functioning were associated with high caregiver burden. While our analyses do not allow us to claim a causal relationship between these variables, we could theorize that unhealthy family dynamics, such as poor communication and hostile interactions, exacerbate the burden perceived by the caregiver. However, it could also be that the experience of burden as a consequence of caring for an individual with schizophrenia leads to a worse family environment. In this regard, other studies have shown that dissatisfaction with family support is linked to caregiver burden and relapse in people with schizophrenia [16, 19, 20]. The present study adds to the growing literature about family functioning as a potential target for treatment that aims to improve schizophrenia outcomes and reduce caregiver burden.

Other factors that were associated with high perceived burden were caregiver hope and caregiver work. We found that lower levels of hope were associated with high caregiver burden. This finding is congruous with previous observations in other populations, such as in informal caregivers of individuals with advanced cancer and multiple sclerosis, respectively [38, 39]. Psychosocial interventions that directly address hopefulness and aim to foster hope among caregivers could be helpful in promoting better outcomes in families of individuals living with schizophrenia. We also found that caregivers that held a job in the past 3 months were more likely to report high burden than those that were not working. This increased level of burden could be explained by distress stemming from having to juggle responsibilities at work and at home and/or increased financial responsibilities related to adult caregiving. Although symptom severity was not identified as a significant variable, we do want to note that the PANSS scores of our study population are on the low side, similar to what has been seen among other treated populations in sub-Saharan Africa [40].

This study had several limitations. The first limitation is the data-driven approach used to dichotomize caregiver burden for statistical purposes. Previous studies have not identified theory-based cut-off points for burden levels using this scale. That said, our cut-off choice was data-driven in that it aligned with a natural break seen in the data. A sensitivity analysis was also performed to address this concern, providing us with more confidence about our interpretation of the findings. Second, given that all participants were solely recruited from outpatient clinics, the sample may not be representative of the larger population of individuals who live with psychotic disorders. This is perhaps also suggested by the relatively higher levels of education of the participants in our study. Many individuals living with schizophrenia in Tanzania may have never accessed formal psychiatric services; therefore, the burden measured in our study may actually be underestimated. It would be reasonable to expect greater perceived burden in the caregivers of those people living with schizophrenia who have never had psychological support nor taken antipsychotic medication.

Another limitation is the cross-sectional design of the study, which does not allow for causal inferences regarding the relationship between family functioning and caregiver burden. An important caveat for interpreting our results is that the effect of family functioning on caregiver burden was not robust to other factors. The estimate changed greatly as other variables were added to the final model, suggesting considerable confounding, and thus, opening the possibility of unmeasured confounding as well. As an exploratory analysis of the relationship between family functioning and caregiver burden, we are not certain that the variables included in the multivariable model are confounders—they may be mediators. Consequently, the possibility that the reported odds ratios are direct effects, instead of total effects, cannot be ruled out. Lastly, the estimated confidence intervals on the odds ratios were wide, particularly for family functioning and caregiver work, indicating a lack of precision in the estimates. It should also be noted that given the high proportion of participants with high caregiver burden (63.1%), the reported odds ratios likely deviated farther from the null than would prevalence ratios for the same association.

In Tanzania, there is legislation in place to protect and promote the rights of individuals with mental disorders. The Persons with Disabilities Act of 2010 covers a wide range of provisions for those with mental illness and/or other disabilities [41]. Of particular importance in the context of this study is that under the Persons with Disabilities Act of 2010, relatives of a person with a disability are obliged to provide social support to such person. Nevertheless, there is no financial social safety net for people with schizophrenia and their families. Our study offers evidence of high levels of perceived burden among informal caregivers in Tanzania and calls for action at the policy and programmatic levels to address the needs of families of individuals with schizophrenia as a more holistic approach to mental health care and support.

## Conclusion

This was the first study to examine levels of perceived burden among caregivers of individuals with schizophrenia and its relationship to family functioning in Tanzania. The established high levels of caregiver burden and association with poor family functioning adds evidence for the need to foster healthy family dynamics in families managing schizophrenia. Future interventions aiming to reduce caregiver burden may benefit from improving family functioning and nurturing hope among caregivers of individuals living with schizophrenia, with special attention for those caregivers who also have formal work responsibilities. Mental health policies and programs should be cognizant of the needs of caregivers so that they can be best positioned as partners in recovery for their relatives living with schizophrenia.

## Supplementary Information

**Additional file 1: Supplemental Table 1.** Sensitivity Analysis. Univariable Regression Models for Caregiver Burden (Cut-Off Point of 32/33) on Characteristics of Individuals with Schizophrenia and Informal Caregivers (*N*=65)

## Data Availability

The dataset used during this current study is available from the corresponding author on reasonable request and with IRB approval.

## Abbreviations

MUHAS: Muhimbili University of Health and Allied Sciences
MNH: Muhimbili National Hospital
MZRH: Mbeya Zonal Referral Hospital
ICD-10: International Classification of Disease
NIMR: National Institute for Medical Research
BAS: Burden Assessment Scale
SCORE-15: Systemic Clinical Outcome and Routine Evaluation
HHI: Hearth Hope Index
DUREL: Duke University Religion Index
IR: intrinsic religiosity
PANSS: Positive and Negative Syndrome Scale
WHODAS 2.0: World Health Organization Disability Assessment Schedule-Second Version
GSE: General Self-Efficacy Scale
ISMI: Internalized Stigma of Mental Illness
PROMIS v2.0: Patient-Reported Outcomes Measurement Information System
NIH: National Institutes of Health
