# Supplementary material for: Perceived burden and family functioning among informal caregivers of individuals living with schizophrenia in Tanzania: a cross-sectional study
Source: BMC Psychiatry. 2022 Jan 4;22:10. doi: 10.1186/s12888-021-03560-0 (PMC8728903; doi:10.1186/s12888-021-03560-0)
Supplement: Supplementary file 1 — Additional file 1: Supplemental Table 1. Sensitivity Analysis. Univariable Regression Models for Caregiver Burden (Cut-Off Point of 32/33) on Characteristics of Individuals with Schizophrenia and Informal Caregivers (N=65) [file 12888_2021_3560_MOESM1_ESM.docx]

**Additional file 1**

| **Supplemental Table 1. Sensitivity Analysis. Univariable Regression Models for Caregiver Burden (Cut-Off Point of 32/33) on Characteristics of Individuals with Schizophrenia and Informal Caregivers (N=65)** | | | | |  |
| --- | --- | --- | --- | --- | --- |
|  |  |  |  |  |  |
|  |  |  | **Univariable Model** | |  |
|  |  |  |  |  |  |
| ***Characteristics of Individuals with Schizophrenia*** | | **N** | **Crude OR (95% CI)** | **p-value** |  |
| **Sex** | |  |  |  |  |
|  | Male | 43 | REF |  |  |
|  | Female | 22 | 2.67 (0.77; 9.28) | 0.123 |  |
| **Relationship Status** | |  |  |  |  |
|  | Partnered, living together | 10 | REF |  |  |
|  | Partnered, not living together | 11 | 4.50 (0.63; 32.29) | 0.135 |  |
|  | Single, not partnered | 44 | 2.38 (0.59; 9.66) | 0.223 |  |
| **Educational Level** | |  |  |  |  |
|  | Primary or less | 24 | REF |  |  |
|  | Secondary or higher | 41 | 0.64 (0.21; 1.98) | 0.442 |  |
| **Worked in the Past 3 Months** | |  |  |  |  |
|  | No | 28 | REF |  |  |
|  | Yes | 37 | 0.22 (0.06; 0.76) | 0.016 |  |
| **Financial Contribution to Household** | |  |  |  |  |
|  | No | 43 | REF |  |  |
|  | Yes | 22 | 0.36 (0.12; 1.09) | 0.071 |  |
| **Age** | | 65 | 0.95 (0.89; 1.02) | 0.160 |  |
| **Length of Illness** | | 65 | 0.99 (0.93; 1.06) | 0.782 |  |
| **Disability (WHODAS)** | | 65 | 1.00 (0.97; 1.03) | 1.000 |  |
| **Self-Efficacy (GSE)** | | 65 | 1.06 (0.97; 1.16) | 0.182 |  |
| **Instrumental Support (PROMIS)** | | 65 | 1.02 (0.98; 1.07) | 0.311 |  |
| **Internalized Stigma (ISMI)** | | 65 | 2.00 (0.66; 6.01) | 0.219 |  |
| **PANSS Positive** | | 65 | 0.91 (0.80; 1.03) | 0.122 |  |
| **PANSS Negative** | | 65 | 0.89 (0.79; 1.00) | 0.046 |  |
| **PANSS General** | | 65 | 0.95 (0.89; 1.02) | 0.146 |  |
| **PANSS Total** | | 65 | 0.97 (0.93; 1.00) | 0.062 |  |
|  |  |  |  |  |  |
| ***Characteristics of Informal Caregivers*** | | **N** | **Crude OR (95% CI)** | **p-value** |  |
| **Sex** | |  |  |  |  |
|  | Male | 22 | REF |  |  |
|  | Female | 43 | 2.01 (0.68; 6.00) | 0.209 |  |
| **Educational Level** | |  |  |  |  |
|  | Primary or less | 41 | REF |  |  |
|  | Secondary or higher | 24 | 1.13 (0.38; 3.38) | 0.830 |  |
| **Worked in the Past 3 Months** | |  |  |  |  |
|  | No | 31 | REF |  |  |
|  | Yes | 34 | 5.44 (1.67; 17.73) | 0.005 |  |
| **Living with Patient** | |  |  |  |  |
|  | No | 10 | REF |  |  |
|  | Yes | 55 | 0.96 (0.22; 4.16) | 0.954 |  |
| **Age** | | 65 | 0.99 (0.95; 1.03) | 0.515 |  |
| **Family Functioning (SCORE-15)** | | 65 | 26.70 (5.52; 129.22) | <0.001 |  |
| **Hope (HHI)** | | 65 | 0.77 (0.70; 0.88) | <0.001 |  |
| **Religiosity (IR Subscale of DUREL)** | | 65 | 0.61 (0.33; 1.14) | 0.121 |  |
| OR = odds ratio; CI = confidence interval.  WHODAS, World Health Organization Disability Assessment Schedule; GSE, General Self-Efficacy scale; PROMIS, Patient-Reported Outcomes Measurement Information System; ISMI, Internalized Stigma of Mental Illness scale; PANSS, Positive and Negative Syndrome Scale; SCORE-15, Systemic Clinical Outcome and Routine Evaluation; HHI, Herth Hope Index; DUREL; Duke University Religion Index | | | | |  |
